# Does Hepatic Steatosis Influence the Detection Rate of Metastases in the Hepatobiliary Phase of Gadoxetic Acid-Enhanced MRI?

**DOI:** 10.3390/jcm10010098

**Published:** 2020-12-30

**Authors:** Ingo G. Steffen, Thomas Weissmann, Jan Holger Rothe, Dominik Geisel, Sascha S. Chopra, Johannes Kahn, Bernd Hamm, Timm Denecke

**Affiliations:** 1 Klinik für Radiologie, Campus Virchow-Klinikum, Charité–Universitätsmedizin Berlin, 13353 Berlin, Germany; ingo.steffen@charite.de (I.G.S.); thomas.weissmann@charite.de (T.W.); jan-holger.rothe@charite.de (J.H.R.); dominik.geisel@charite.de (D.G.); johannes.kahn@charite.de (J.K.); bernd.hamm@charite.de (B.H.); 2 Klinik für Allgemein-Viszeral-und Transplantationschirurgie, Campus Virchow-Klinikum, Charité–Universitätsmedizin Berlin, 13353 Berlin, Germany; sascha.chopra@charite.de; 3 Klinik und Poliklinik für Diagnostische und Interventionelle Radiologie, Universitätsklinikum Leipzig, 04103 Leipzig, Germany

**Keywords:** magnetic resonance imaging, MRI, gadolinium, gadoxetic acid, hepatic signal fat fraction, liver steatosis

## Abstract

The aim of this exploratory study was to evaluate the influence of hepatic steatosis on the detection rate of metastases in gadoxetic acid-enhanced liver magnetic resonance imaging (MRI). A total of 50 patients who underwent gadoxetic acid-enhanced MRI (unenhanced T1w in- and opposed-phase, T2w fat sat, unenhanced 3D-T1w fat sat and 3-phase dynamic contrast-enhanced (uDP), 3D-T1w fat sat hepatobiliary phase (HP)) were retrospectively included. Two blinded observers (O1/O2) independently assessed the images to determine the detection rate in uDP and HP. The hepatic signal fat fraction (HSFF) was determined as the relative signal intensity reduction in liver parenchyma from in- to opposed-phase images. A total of 451 liver metastases were detected (O1/O2, *n* = 447/411). O1/O2 detected 10.9%/9.3% of lesions exclusively in uDP and 20.2%/15.5% exclusively in HP. Lesions detected exclusively in uDP were significantly associated with a larger HSFF (area under curve (AUC) of receiver operating characteristic (ROC) analysis, 0.93; *p* < 0.001; cutoff, 41.5%). The exclusively HP-positive lesions were significantly associated with a smaller diameter (ROC-AUC, 0.82; *p* < 0.001; cutoff, 5 mm) and a smaller HSFF (ROC-AUC, 0.61; *p* < 0.001; cutoff, 13.3%). Gadoxetic acid imaging has the advantage of detecting small occult metastatic liver lesions in the HP. However, using non-optimized standard fat-saturated 3D-T1w protocols, severe steatosis (HSFF > 30%) is a potential pitfall for the detection of metastases in HP.

## 1. Introduction

Non-alcoholic fatty liver disease (NAFLD), ranging from simple steatosis to non-alcoholic steatohepatitis (NASH) is considered to be the hepatic manifestation of the metabolic syndrome and has become the leading chronic liver disorder in the developed world with an estimated global prevalence up to 30% [1,2,3]. Liver steatosis or even steatohepatitis has also been reported to be associated with various chemotherapeutic regimens [4,5,6]. Thus, the presence of hepatic steatosis is very common in patients undergoing liver imaging and can affect accurate lesion detection by altering parenchymal appearance and lesion-to-background contrast [7,8].

In recent years, the hepatocyte-specific contrast agent gadoxetic acid has been increasingly and successfully used to enhance the sensitivity of magnetic resonance imaging (MRI) in the detection of malignant liver lesions [9,10,11]. A gadoxetic acid-enhanced MRI protocol comprises all conventional sequences used for standard liver MRI workup, which are unenhanced T1w and T2w sequences and a first-pass dynamic contrast-enhanced series (arterial, portal-venous, venous, and equilibrium phase). Compared with conventional liver MRI, the strong specific hepatocellular uptake of gadoxetic acid results in a hepatobiliary plateau phase with an increase in signal intensity (SI) in the hepatic parenchyma, thereby improving contrast relative to nonhepatocellular tissues without specific gadoxetic acid uptake such as liver metastases [12]. The recommended and internationally accepted MRI protocol with gadoxetic acid consists of axial gapless three-dimensional interpolated T1-weighted fat-saturated breath-hold gradient echo sequences with a flip angle of 10°–12° (3D-T1w-FS; e.g., VIBE (Siemens), THRIVE (Philips), LAVA (GE) for dynamic (arterial to equilibrium phase) and hepatobiliary phase (delay, 10–20 min) imaging [10,13]. These robust sequences require only a short breath-hold interval and have high spatial resolution (slice thickness, 2–3 mm). In patients with severe hepatic steatosis, however, the gain in signal in the hepatobiliary phase is counteracted by signal suppression in the steatotic liver parenchyma due to the fat saturation impulse of the 3D-T1w-FS sequence. This could potentially impair lesion detection by reducing the SI contrast between the hepatocellular parenchyma and the metastatic tissue.

The aim of this study was to analyze the effect of hepatic steatosis on the detection rate of metastases in fatty livers in patients examined using a standard gadoxetic acid-enhanced liver MRI protocol.

## 2. Materials and Methods

### 2.1. Patients

Inclusion criteria were a gadoxetic acid-enhanced liver MRI in our institution with at least one histologically confirmed liver metastasis and the presence of hepatic steatosis >10% based on chemical shift method. Patients with hemochromatosis as well as hemosiderosis were excluded to avoid T1 signal intensity alteration due to iron overload. Patients with glomerular filtration rate (GFR) <30 mL/min/1.73 m were excluded to avoid impairment by abnormally high excretion rates of the liver. Further exclusion criteria were a serum bilirubin level >2.0 mg/dL and patient age under 18 years. By applying these criteria to our institutional database using a 37-month period, a total of fifty patients (24 men, 26 women; age between 33 and 80 years, median: 62.9 years) were consecutively included from our institutional database. Primary tumors were distributed as follows: colorectal adenocarcinoma (*n* = 21), neuroendocrine tumors (*n* = 11), breast cancer (*n* = 9), pancreatic adenocarcinoma (*n* = 5), ovarian cancer (*n* = 2), choroid melanoma (*n* = 1), and urothelial carcinoma (*n* = 1). Systemic chemotherapy during or up to 30 days before the study period was performed in 19 patients.

### 2.2. Magnetic Resonance Imaging

All MRI examinations were performed at 1.5 Tesla: six patients were examined with a Signa Excite system (GE Healthcare, Milwaukee, Wisconsin, USA) and 44 patients with a Philips Gyroscan ACS-NT MRI system (Philips Medical Systems, Best, The Netherlands) using phased-array surface coils. All examinations were performed using a standard protocol for liver workup including unenhanced gradient echo (GRE) sequence, fat-saturated contrast-enhanced images, and T2w turbo spin echo (TSE) images in axial orientation (uDP, unenhanced + early dynamic phase sequences) and hepatobiliary late-phase sequences (HP) according to the terminology proposed by an expert consensus panel [14]. The late phase was acquired using a fat-saturated LAVA (liver volume acquisition with volume acceleration) or a THRIVE (T1 high-resolution isotropic volume examination) in the transverse and coronal planes 20 min after contrast agent injection. All patients were administered a standard dose of gadoxetic acid (Gd-EOB-DTPA, Primovist/Eovist, Bayer HealthCare Pharmaceuticals, Berlin, Germany) of 0.025 mmol/kg body weight as an intravenous injection at a flow rate of 1–2 mL/s, followed by a 20 mL saline flush.

The MRI protocol on the Signa Excite consisted of the following: T1w-GRE in/opposed phase (TR/TE: in phase 160 ms/4.4 ms, opposed phase 160 ms/2.2 ms, 60° flip angle, 256 × 224 matrix, 6 mm slice thickness), LAVA unenhanced, triphasic dynamic and as HP (TR/TE, 4.5 ms/2.1 ms; 12° flip angle; 320 × 192 matrix), T2w TSE (TR/TE, 2100 ms/101 ms; 90° flip angle; 320 × 256 matrix; 6 mm slice thickness), fat-saturated T2w TSE (TR/TE, 2400 ms/71 ms, 90° flip angle; 320 × 256 matrix; 6 mm slice thickness).

The protocol on the Gyroscan included the following: T1w GRE in/opposed phase (TR/TE, in phase 174 ms/4.6ms, opposed phase 174 ms/2.3 ms; 80° flip angle; 256 × 179 matrix; 6 mm slice thickness), THRIVE unenhanced, triphasic dynamic after contrast agent administration and as late phase (TR/TE, 4.0 ms/1.9 ms, 10° flip angle, 192 × 192 matrix, 5 mm slice thickness), T2w TSE (TR/TE: 1600 ms/100 ms, 90° flip angle, 400 × 299 matrix, 6 mm slice thickness), fat-saturated T2wTSE (TR/TE 2100 ms/90 ms, 90° flip angle, 272 × 192 matrix, 6 mm slice thickness).

### 2.3. Image Analysis

**Figure 1:**
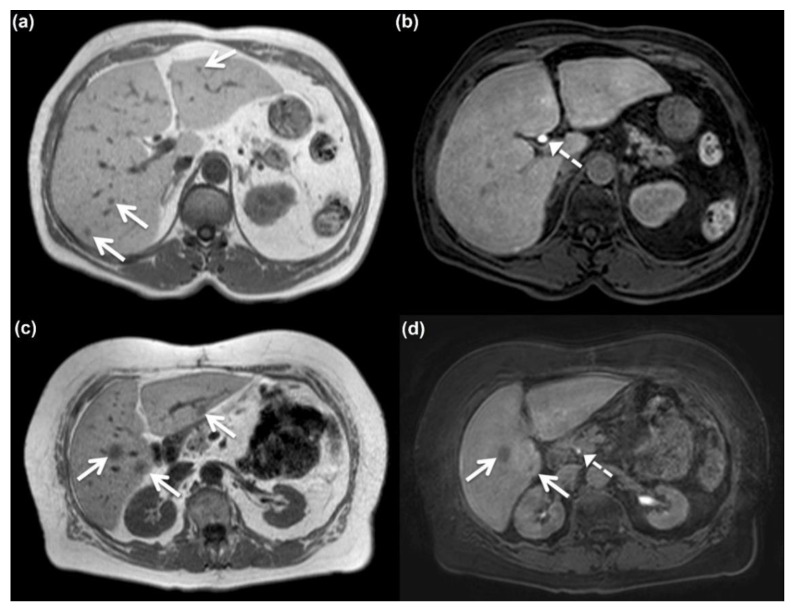
Patient 1 (**a**,**b**) and patient 2 (**c**,**d**), both with fatty liver and metastatic lesions (arrows) seen on unenhanced T1-weighted non-fat-saturated images (**a**,**c**) but only partially positive on the corresponding hepatobiliary phase images (**b**,**d**) acquired after gadoxetic acid administration. Note biliary excretion of the contrast agent (broken arrow).

Image analysis was performed with a Centricity RA 1000 workstation from GE Medical Systems. The hepatic signal fat fraction (HSFF) [15] was measured and averaged across three regions of interest (ROIs; left, middle, right) placed in the hepatic parenchyma on a representative mid-hepatic section in the in and opposed phase (HSFF, (SI_(opposed)_ − SI_(in)_)/2 × SI_(in)_) as was gadoxetic acid uptake using unenhanced and HP images (gadoxetic acid uptake, (SI_(contrast enhanced)_ − SI_(unenhanced)_)/SI_(unenhanced)_). Images were reviewed for the presence of liver metastases by two independent observers (O1/O2) with different levels of experience in liver imaging (observer 1, 9 years; observer 2, 3 years). Observers were blinded to clinical and patient information. uDP images and HP images were presented separately in randomized order and at least 4 weeks apart to avoid memory bias. All lesions with metastatic appearance (uDP, T1w in-phase hypointense, T2w hyperintense but not benign cyst, contrast behavior different from hemangioma; HP, hypointense) were documented. For HP images, after complete analysis and documentation, T2w images were provided to remove benign cysts from the chart. For each metastatic lesion identified, the reader recorded its segmental location, largest diameter, and distance from the liver surface. Subsequently both datasets were automatically merged using lesion characteristics. In case of location/diameter deviations greater than 3 mm, lesions were manually matched in a consensus reading. An example of two patients with fatty liver and metastatic lesions visible on unenhanced T1-weighted non-fat-saturated images but only partially positive on the corresponding hepatobiliary phase images is illustrated in Figure 1.

### 2.4. Statistical Analysis

Data were analyzed using the R software (Version 4.0.2; R Foundation for Statistical Computing, Vienna, Austria). Based on histograms and the Shapiro–Wilks test, we assumed nonnormal distribution of data, and metric parameters were described by median, interquartile range (IQR; 25th–75th percentile), and range (min–max). The association of two dichotomous parameters was analyzed using contingency tables and the chi-square test. In the case of absolute frequencies in contingency tables of less than five, Fisher’s exact test was performed. Paired binary data were analyzed using the McNemar test. Receiver operating characteristics (ROC) analysis was performed to analyze the association of lesion diameter and HSFF with the detectability of lesions. The sensitivity and specificity calculated using the ROC analysis is based on the following definitions: positive = uDP^+^/HP^−^ (uDP^−^/HP^+^), negative = rest of cases. The optimal threshold was determined using the maximum Youden index (sensitivity + specificity − 1). A logistic regression model was used to analyze the detectability of lesions in relation to lesion diameter, HSFF, and observer. The results of the logistic regression model were displayed using probability plots. Interobserver agreement was quantified using Cohen’s kappa. All tests were two-sided, and the significance level was set to α = 0.05.

## 3. Results

### 3.1. Detected Lesions

The two observers detected a total of 451 metastases (O1/O2, *n* = 447/411) with diameters ranging from 2 to 105 mm (O1/O2: median (IQR), 7 (5–12)/8 (5–12) mm). The rate of metastatic lesions identified in both unenhanced and dynamic sequences (uDP) and hepatobiliary late-phase sequences (HP) was 69.0%/75.2% for O1/O2 (Table 1). The rate of metastatic lesions visible only in HP was significantly higher (O1/O2, 20.2%/15.5%) compared to lesions visible only in uDP (O1, O2, 10.9%/9.3%; McNemar test, both *p* < 0.01). In a patient-based analysis, lesions visible only in HP occurred in 22/23 patients (44%/46%, O1/O2) whereas lesions visible exclusively in uDP were present in 5 patients (10% both observers).

### 3.2. Interobserver Differences

The rate of discordant findings for O1 vs. O2 was 4.2% in uDP and 8.2% in HP (Table 2). Kappa statistics showed excellent agreement of observers in uDP (Cohen’s kappa = 0.88) and substantial agreement of observers in HP (Cohen’s kappa = 0.68).

### 3.3. Lesions Detected Exclusively in the Hepatobiliary Phase

**Figure 2:**
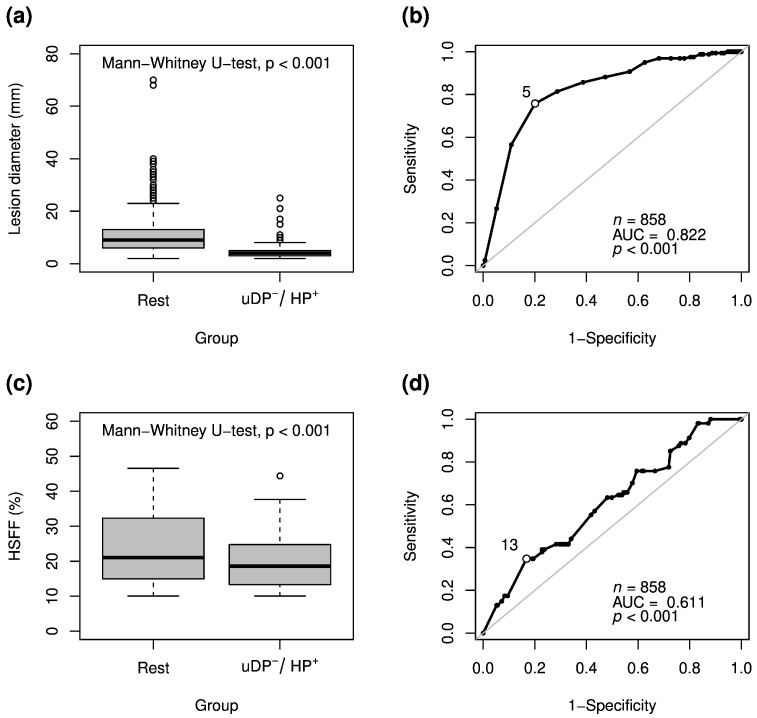
Boxplots (**a**,**c**) and receiver operating characteristic (ROC) analysis (**b**,**d**) for metastatic lesions detected exclusively in the hepatobiliary phase images but missed in the unenhanced and dynamic phase images (uDP^−^/HP^+^) in relation to lesion diameter and HSFF for both observers. The circle with white background (**b**,**d**) indicates the optimal cutoff (based on Youden index).

Metastatic lesions exclusively positive in HP were significantly smaller than all other detected lesions (median (IQR), 4 (3–5) mm vs. 9 (6–13) mm; *p* < 0.001; Figure 2a). ROC analysis showed an AUC (area under curve) of 0.82 (*p* < 0.001) with an optimal cutoff (Youden index) of 5 mm (Figure 2b). The case “lesion is positive in HP and negative in uDP” was more likely to occur with a lower HSFF (median (IQR), 19 (13–25)% vs. 21 (15–32)%; *p* < 0.001; Figure 2c). In ROC analysis the AUC was 0.61 (*p* < 0.001) with an optimal cutoff (Youden index) of 13% HSFF (*p* < 0.001; Figure 2d). 

Total serum bilirubin levels ranged from 0.2 to 2.0 mg/dL. No significant differences were observed between lesions visible only in HP images (median (IQR), 0.4 (0.3–1.2) mg/dL vs. 0.4 (0.3–0.7) mg/dL; *p* = 0.23). The observed median relative gadoxetic acid uptake ranged from 46% to 130%. Metastases observed only in HP images showed no significant differences to other lesions (median (IQR), 89 (78–106)% vs. 91 (77–103)%; *p* = 0.61). 

### 3.4. Lesions Detected Exclusively in the Unenhanced and Dynamic Phases

**Figure 3:**
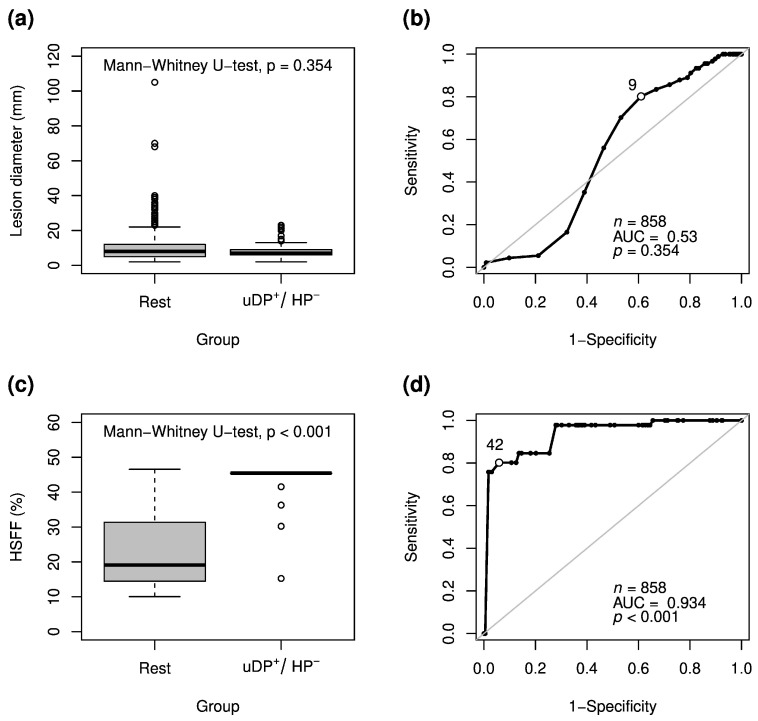
Boxplots (**a**,**c**) and ROC analysis (**b**,**d**) for metastatic lesions detected exclusively in unenhanced and dynamic phase images but missed in hepatobiliary phase images (uDP^+^/HP^−^) in relation to lesion diameter and HSFF for both observers. The optimal cutoff according to Youden index is marked by a circle with white background (**b**,**d**).

Metastatic lesions exclusively positive in uDP were similar in size to all other detected lesions (median [IQR], 7 (6–9) mm vs. 8 (5–12) mm; *p* = 0.354) with an AUC of 0.53 (*p* = 0.354) in ROC analysis (Figure 3a,b). The case “lesion is positive in uDP and negative in HP” was more likely to occur with a higher HSFF (median (IQR), 45 (45–45)% vs. 19 (15–31)%; *p* < 0.001; Figure 3c). ROC analysis for lesions detected only in uDP sequences but missed in the HP revealed an AUC of 0.93 with an optimal HSFF cutoff (Youden index) of 42% (sensitivity, 80.2%; specificity, 94.1%; *p* < 0.001; Figure 3d). The cutoff for HSFF closest to 100% sensitivity with acceptable specificity was 30% (98% sensitivity; 72% specificity). 

No significant differences were observed for serum bilirubin levels between lesions visible only in uDP images (median (IQR), 0.4 (0.4–0.4) mg/dL vs. 0.4 (0.3–0.7) mg/dL; *p* = 0.41). Metastases observed only in uDP images showed no significant differences in relative gadoxetic acid uptake compared to other lesions (median (IQR), 96 (96–96)% vs. 87 (77–106)%; *p* = 0.64). 

### 3.5. Multivariable Logistic Regression Model

**Figure 4:**
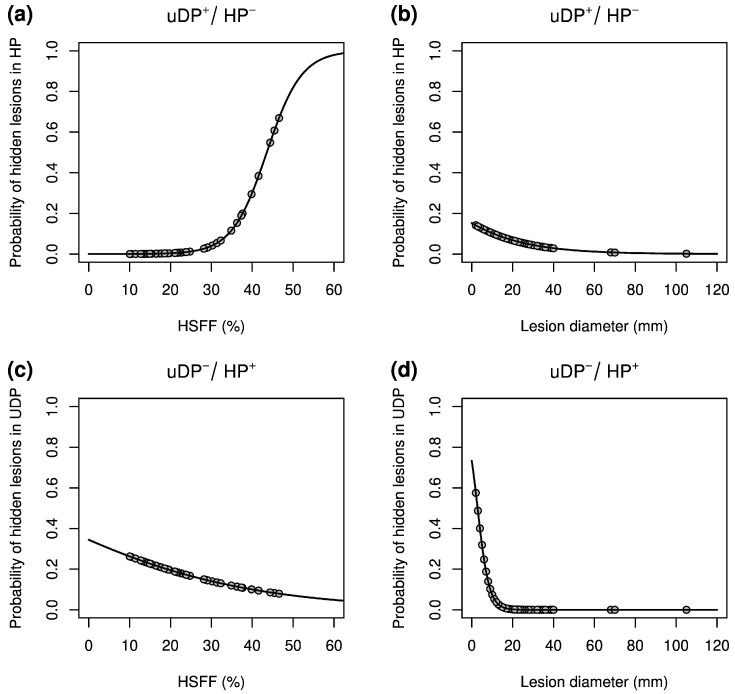
The probability of lesions visible exclusively in the unenhanced and dynamic phase images (uDP^+^/HP^−^) notably increases with the hepatic signal fat fraction (HSFF), beginning at about 30% (**a**). This phenomenon tends to occur predominantly in small lesions (**b**). In contrast, the probability for lesions visible exclusively in the hepatobiliary phase images (uDP^−^/HP^+^) increases as the HSFF decreases (**c**) and peaks for lesions with diameters smaller than approximately 20 mm (**d**).

A multivariable logistic regression model showed that failure to detect lesions in HP was significantly associated with a higher HSFF (*p* < 0.001) whereas a tendency toward significance was found for lesion diameter (*p* = 0.08), and the observer had no significant influence (*p* = 0.83). The probability of lesions that were detected exclusively in uDP in association with the hepatic signal fat fraction and diameter according to the logistic regression model is depicted in Figure 4a,b. The detection of metastases observed exclusively in HP was significantly associated with a smaller lesion diameter (*p* < 0.001) and a smaller HSFF (*p* < 0.001) whereas the observer had no significant influence (*p* = 0.99). The probability of lesions that were detected exclusively in HP in association with the hepatic signal fat fraction and diameter according to the logistic regression model is depicted in Figure 4c,d.

## 4. Discussion

The hepatobiliary contrast agent gadoxetic acid allows combined dynamic contrast-enhanced imaging and hepatocyte-specific imaging in one examination [11] and has been proven to enable highly efficacious detection and characterization of focal liver lesions with a corresponding impact on therapeutic management [9,10,11,16,17]. This study confirms the contribution of the HP in lesion detection with approximately 20% of metastatic lesions (especially small lesions) only seen in this contrast phase. However, our results also indicate that the efficacy of gadoxetic acid is reduced when standard fat-saturated 3D-T1w sequences are used in severe hepatic steatosis, with approximately 10% of lesions being seen only in unenhanced and dynamic images in patients with a large HSFF (relative signal drop from in to opposed phase).

The high accuracy of gadoxetic acid-enhanced MRI for preoperative liver imaging and for patients with suspected hepatic malignancy has been demonstrated in several studies [12,18,19]. A prospective randomized multicenter trial in 342 patients with suspected colorectal liver metastases demonstrated the highest number of correctly identified metastases in resected liver parts confirmed by histopathology for gadoxetic acid-enhanced MRI (88%) compared to MRI with extracellular contrast medium (74%) and contrast-enhanced computed tomography (CT, 62%) [11]. Additionally, gadoxetic acid-enhanced MRI had the highest true-positive detection rate for liver metastases on a patient-to-patient basis upon first examination (gadoxetic acid MRI, 70% vs. MRI with extracellular contrast medium (ECCM-MRI), 64% vs. CT, 59%) and the lowest rate of intraoperative change of surgical strategy (gadoxetic acid MRI, 28%; ECCM-MRI, 32%; CT, 47%). The additional HP of the gadoxetic acid MRI protocol enables imaging of the liver during a long plateau phase with sufficient hepatocellular uptake for optimal contrast between surrounding parenchyma and malignant lesions. Sufficient uptake is reached as early as 10–20 min after contrast agent injection [20]. Even with standard MRI devices, regardless of the manufacturer, it is possible to achieve good image quality with the very robust 3D T1w fat-saturated breath-hold sequence with a small slice thickness and short breath-hold intervals (approximately 20 s). Because of this long plateau of the HP, this sequence can be repeated and, if needed, slightly modified regarding acceleration techniques and slice thickness as well as *z*-axis coverage. An international expert consensus conference in 2009, therefore, recommended to use such a sequence for HP imaging, and this general recommendation has not changed since then as it enables the detection of sub-centimeter nodules [21]. The present study clearly confirms the advantage of additional HP images, as the two observers detected 15–20% of lesions, especially small ones, only in HP images and not with the uDP sequences. 

Since the recommended standard sequence of the gadoxetic acid protocol, the 3D-T1w-FS, is a fat-saturated sequence, it is imaginable that the increase in parenchymal SI is reduced by fat suppression when steatosis is present. A recent study [22,23] used exactly this standard sequence (VIBE) along with the conventional uDP sequences and diffusion-weighted imaging (DWI) on a 3T MRI system in 23 patients with 31 metastases ≤1 cm and found a superior preoperative lesion detection rate of MRI (93%) vs. multidetector CT (43%). Two small lesions missed by MRI could be attributed to the described phenomenon of HP-negative lesions due to steatosis. A separate analysis of detection rates for uDP, DWI, and HP was not done in this study and, therefore, we do not know whether uDP or DWI might have detected the two lesions missing in HP images. In the present study, the approximately 10% of lesions only detected in uDP images but not in HP images were significantly associated with a larger HSFF. While uDP compensated for the inability of the HP to detect these lesions in our study setting, it is imaginable in view of the report of Berger-Kulemann et al. [22] that, with a proper reference standard, there might have been even more lesions negative on HP but also negative on uDP. This is why considerations on the examination protocol are important to avoid the HP-negative phenomenon in fatty liver. 

Strategies to overcome this problem would be to change the contrast agent to ECCM or to invest extra time in high-quality DWI, an additional T1w sequence without fat saturation, or a 3D-T1w-FS sequence with a larger flip angle. All of these solutions have potential disadvantages such as longer acquisition time through additional sequences or inferior results to be expected in comparison with optimal HP images (DWI, ECCM) [11,23]. Therefore, it would be desirable to undertake such steps only in patients with a high risk of having lesions expected to be missed by standard gadoxetic acid MRI because of steatosis. The present study revealed a cutoff of about 30% HSFF (98% sensitivity, 72% specificity) derived from in-/opposed-phase imaging, usually acquired at the beginning of a liver MRI examination. The MRI protocol could thus be adjusted in patients with a higher HSFF.

It can be argued whether there are other mechanisms diminishing the gain in T1-SI of steatotic parenchyma such as space-occupying fat vacuoles, lesser number/density of hepatocytes due to the underlying hepatic disease (e.g., NASH), and reduced hepatocyte function regarding expression of transporter molecules on the cell surface, which are also responsible for gadoxetic acid uptake. A study by Onishi et al. investigated these possible mechanisms using the same protocol as in our study in 166 patients, 52 of them with steatosis [24]. While liver/spleen contrast was inversely correlated with the fat fraction, relative enhancement ratios of liver and spleen did not differ between steatotic and non-steatotic patients. This explains the rather good hepatic enhancement (gadoxetic acid uptake) measured in our patients, ranging from 46% to 130%, and also why it was not linked with the phenomenon of HP-negative lesions [24]. Furthermore, bilirubin is a known competitor of gadoxetic acid at the membrane transporter of hepatocytes and also a marker of impaired liver function [25,26]. Therefore, one exclusion criterion in this study was a total bilirubin >2 mg/dL.

In this study, the standard approved dose for gadoxetic acid (0.025 mmol/kg) was used. The inferiority of this relatively low dose, especially for dynamic-phase imaging has been stated [27] and the use of 0.05 mmol/kg (off label use) might be discussed as an alternative to address the issue of lesion conspicuity in the hepatobiliary phase. However, it has been recommended to take caution when using gadolinium-based contrast agents and use the lowest doses possible for reducing gadolinium accumulation in brain [28].

This retrospective single-center study has several limitations. First, the hepatic signal fat fraction is less accurate than histopathology [15] and impairment of quantification by confounding factors such as fibrosis or iron deposition cannot be ruled out. It has to be stated clearly that HSFF is a suboptimal technique for quantifying hepatic steatosis, which might however be acceptable in this study to prove the concept. Nevertheless, chemical-shift-based liver imaging is an accepted technique for assessing hepatic steatosis and confirmed by correlation with fine-needle aspiration cytology in several studies [29,30]. Only patients with an increased degree of liver steatosis were included in this study. This might be tolerable due to the exploratory character of this study. However, a confirmatory trial should also include a control group without liver steatosis. In this study, we did not analyze DWI, which would have improved the standard of reference for the HP. Additional DWI might even increase the number of false-negative lesions on HP. Histopathological confirmation was only available for at least one hepatic metastasis per patient but not for all lesions. Thus, lesions seen exclusively on the hepatobiliary phase could be benign as well. As lesions from multiple different types of cancer were included in this study, the enhancement characteristics of many of these cancers can be significantly different among each other. A subgroup analysis (results not shown) indicates that the association of hidden lesions in HP and hepatic steatosis might be influenced by the type of cancer. Finally, hepatobiliary late-phase images in this study were acquired at a low flip angle. However, increasing the flip angle in hepatocyte-phase MRI with gadoxetic acid has been shown to improve hypointense lesion detection and conspicuity, particularly for small lesions [31]. Optimized protocols might be helpful in overcoming the potential pitfall of hidden lesions in hepatobiliary late-phase images.

## 5. Conclusions

Our study confirms the advantage of gadoxetic acid-enhanced MRI, namely the detection of small, otherwise occult metastatic liver lesions in the HP. However, using older non-optimized standard fat-saturated 3D T1w protocols, severe steatosis (HSFF > 30%) is a potential pitfall for the detection of metastases in hepatobiliary late-phase images. The use of optimized protocols may avoid this important pitfall.

## Figures and Tables

**Table 1:** Contingency table for the detection of metastatic lesions in unenhanced and dynamic phase (uDP) vs. hepatobiliary phase (HP) images by observer.

	uDP	HP		
		Not Detected	Detected	Total
Observer 1	Not detected	4 (0.9%)	91 (20.2%)	95 (21.1%)
	Detected	49 (10.9%)	307 (68.0%)	356 (78.9%)
	Total	53 (11.8%)	398 (88.2%)	451 (100.0%)
Observer 2	Not detected	40 (8.9%)	70 (15.5%)	110 (24.4%)
	Detected	42 (9.3%)	299 (66.3%)	341 (75.6%)
	Total	82 (18.2%)	369 (81.8%)	451 (100.0%)

**Table 2:** Contingency table for the detection of metastatic lesions by observer 2 vs. observer 1 separately for unenhanced and dynamic phase (uDP) and hepatobiliary phase (HP) images.

	Observer 1	Observer 2		
		Not Detected	Detected	Total
uDP	Not detected	93 (20.6%)	2 (0.4%)	95 (21.0%)
	Detected	17 (3.8%)	339 (75.2%)	356 (79.0%)
	Total	110 (24.4%)	341 (75.6%)	451 (100%)
HP	Not detected	49 (10.9%)	4 (0.9%)	53 (11.8%)
	Detected	33 (7.3%)	365 (80.9%)	398 (88.2%)
	Total	82 (18.2%)	369 (81.8%)	451 (100%)

## Data Availability

Data available on request due to privacy/ethical restrictions.
